# Seat Assignments With Physical Distancing in Single-Destination Public Transit Settings

**DOI:** 10.1109/ACCESS.2021.3065298

**Published:** 2021-03-10

**Authors:** Jane F. Moore, Arthur Carvalho, Gerard A. Davis, Yousif Abulhassan, Fadel M. Megahed

**Affiliations:** Farmer School of BusinessMiami University 6403 Oxford OH 45056 USA; Department of Industrial and Systems EngineeringAuburn University 1383 Auburn AL 36849 USA; Department of Occupational Safety and HealthMurray State University 5675 Murray KY 42071 USA

**Keywords:** Airplane boarding, COVID-19, mixed integer programming (MIP) model, operations research, prescriptive analytics, public transport, school bus seating

## Abstract

While the importance of physical (social) distancing in reducing the spread of COVID-19 has been well-documented, implementing similar controls in public transit remains an open question. For instance, in the United States, guidance for maximum seating capacity in single-destination public transit settings, such as school buses, is only dependent on the physical distance between passengers. In our estimation, the available models/guidance are suboptimal/inefficient since they do not account for the possibility of passengers being from the same household. This paper discusses and addresses the aforementioned limitation through two types of physical distancing models. First, a mixed-integer programming model is used to assign passengers to seats based on the reported configuration of the vehicle and desired physical distancing requirement. In the second model, we present a heuristic that allows for household grouping. Through several illustrative scenarios, we show that seating assignments can be generated in near real-time, and the household grouping heuristic increases the capacity of the transit vehicles (e.g., airplanes, school buses, and trains) without increasing the risk of infection. A running application and its source code are available to the public to facilitate adoption and to encourage enhancements.

## I. Introduction

Over the past year, the incidence of COVID-19 (caused by SARS-CoV-2) has increased exponentially, resulting in 91.9 million confirmed cases and 1.97 million deaths worldwide as of January 13, 2021 [1]. While several vaccines have been developed, their availability has been hampered by: (a) supply limitations, where the available supply of vaccines is much less than the worldwide demand, which is expected to disproportionately affect the roll out of vaccines in economically disadvantaged communities and developing nations [2]; (b) logistical constraints [3], e.g., the vaccine developed by Pfizer and BioNTech requires storage at −70°C ± 10°C [4]; and (c) existing vaccines have not been approved for children and pregnant women. Hence, the continued utilization of non-pharmaceutical countermeasures (e.g., hand-washing, face coverings, and physical distancing) is expected for the near future [5].

Governments have resorted to different strategies and restrictions to combat the COVID-19 pandemic, which have resulted in an estimated 4.3% decline in the worldwide GDP when compared to 2019 [6]. The lockdowns, physical distancing requirements, loss of loved ones and jobs, and the alteration of daily routines have resulted in detrimental mental health outcomes [7]. To balance the health risks with economic/educational incentives, several governments have allowed the continuation of some critical services provided by schools, businesses, and/or transportation companies during the pandemic [8]–[10].

Public transit plays an important role in transporting people/pupils to work/school. This paper addresses the seating assignment problem, while enforcing physical distancing, in single-destination public transit settings. In particular, we focus on the subset of single-destination public transit settings where the list of passengers are known in advance (e.g., school/employee buses, airlines, and trains).

Existing approaches to seat assignment with physical distancing vary in complexity. Several airlines have implemented policies for boarding, seating, and deboarding [11]. For example, Delta Air Lines has implemented the policy of blocking middle seats. Moreover, the company is boarding all their flights from back to front [12]. An unintended consequence of this policy is that the physical distancing between passengers varies according to the aircraft’s size. School bus operators often resort to more granular policies, where seating capacity is constrained by the school bus seating configuration and the required physical distance between passengers [13]. Within the above context, Salari *et al.* [11] present two mixed integer programming (MIP) models for seat assignment: (a) a model focusing on seating passengers far enough away from each other, and (b) a model focusing on the distance between assigned seats and the aisle since aircrew members walk down the aisle during flight. Note that similar MIP-based formulations have been developed for seat assignments during the COVID-19 pandemic for non-transportation settings (e.g., see [14]–[16]).

While the highlighted policies and literature address some aspects of physical distancing, they are suboptimal in practice. In our estimation, their limitations can be attributed to two main reasons. First, none of the aforementioned methods have explicitly accounted for household relationships among passengers/individuals. It is well-documented that individuals from the same household do not need to practice physical distancing [9]. Hence, ignoring household grouping unnecessarily reduces the capacity of the vehicle by requiring physical distancing between individuals from the same household (i.e., the practice increases the inefficiency of the transit). Second, the above seating assignment models do not consider boarding order, which should complement an optimal seating assignment strategy to minimize the likelihood of spreading COVID-19 [17]–[20].

The overarching goals of this paper are to develop appropriate mathematical models alongside a publicly available web application that can be used by practitioners while overcoming the limitations mentioned above. Specifically, our developed application utilizes and extends existing prescriptive analytics techniques/models to automate and optimize the seating problem accounting for different vehicle configurations and physical distancing requirements while providing flexibility to co-seat passengers from the same household. The graphical user interface developed as part of our application allows coordinators to easily input the data required by the models, namely: (a) a vehicle configuration; (b) the desired physical distancing; (c) a definition of groups if at least 2 passengers belong to the same household; and (d) the passenger boarding order, e.g., as determined by the bus route in a school bus schedule. The result from our application is a seating chart that can be communicated/shared with the appropriate personnel.

To maximize the utility and usability of our application as well as to support our discussion in this paper on the importance of considering groups, we allow the users of our application to consider two different scenarios. The first scenario ignores grouping of passengers, which provides the MIP programming solution for the non-grouped seating assignment problem. This solution translates the existing literature [15] to practice. The attained solution provides an “optimal” solution if household relationships are ignored, i.e., an upper bound for the number of seats that can be filled given a layout and a physical distancing threshold. However, this solution is suboptimal in practical cases, where physical distancing tend to be ignored for members of the same household. Hence, in the second scenario, we propose a heuristic that allows for seat assignment with grouping based on a specified boarding order, vehicle configuration, and physical distancing requirement.

**FIGURE 1.**
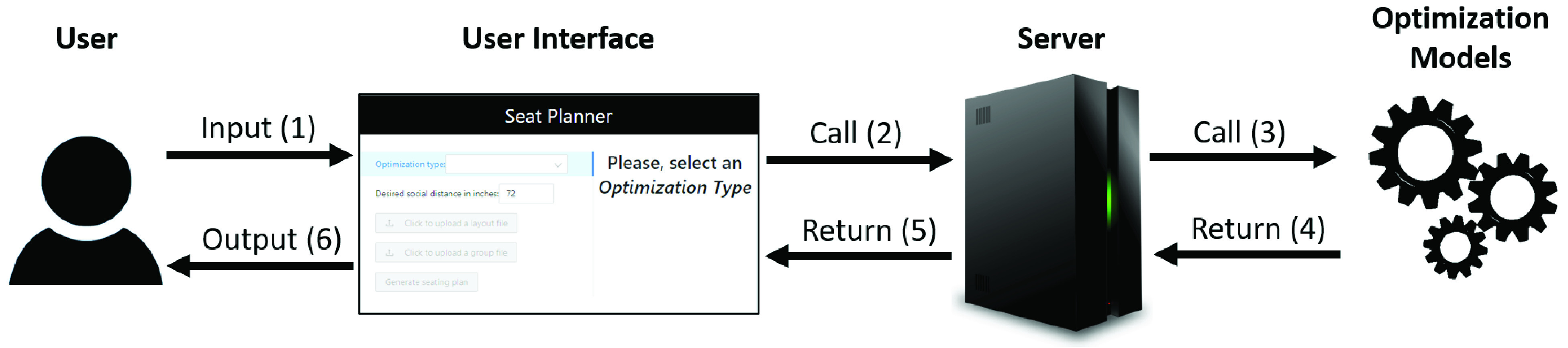
Bird’s-eye view of the proposed application. The numbers in parentheses represent the execution order.

To summarize, we have developed an application and the underlying formulae in an effort to determining optimal seating arrangements and, thus, to promote efficiency while practicing effective measures for preventing the spread of COVID-19. As Fig. 1 illustrates, through the use of the developed decision support system, a data engine brings data into the application, an optimization engine performs calculations based on which of the two models was selected and, finally, information about the optimal seating chart is shared with the user in a comprehensive format. The two models we consider are required to highlight that the relationship among passengers may play a significant role in determining the optimal seating arrangement, as couples or family members may sit in close proximity to each other. This would not be the case for strangers during times of necessary physical distancing, such as during the COVID-19 pandemic.

Besides this introductory section, the rest of the paper is organized as follows. Section II provides detailed information pertaining to the mathematical models used by our application, i.e., the MIP model and a heuristic formulation that takes into account the ordering and grouping of passengers. In Section III, we describe how a user can provide the input required by our models as well as what the resulting output looks like. Then, we describe several examples of how the application can be utilized in Section IV. Finally, Section V describes the implications of our experimental results to advancing research/practice, and it suggests opportunities for future research. In the appendix, we provide a link to our running application to encourage its use by practitioners. Furthermore, we share a link to the application’s source code to facilitate its adoption and the extension of our work in future research.

## II. Optimization Models

In the following subsections, we introduce the two models used by our application.

### A. MIP Formulation When no Household/Grouping Information is Available

In the case when no household/grouping information is available to the transit operator or, alternatively, no passengers belong to the same household, we adopt the algorithmic approach presented in [15] in the context of classroom seat assignments to our single-destination mass transit problem. The approach presented in [15] can be formulated as a MIP problem. As with any optimization problem, our formulation is comprised of input parameters, decision variables, objective functions, and mathematical constraints. These components are described in what follows.

#### Input Parameters

The input represents fixed data needed for the MIP model’s formulation. In particular, the following two inputs are required by the MIP model:

(a) A physical distancing minimum threshold (in inches). This input is used internally by the application to compute a minimum distance matrix between the different passengers, which is used to ensure that the distance between any pair of passengers abides by the physical distancing threshold.
(b) The vehicle’s layout, where the (x,y) coordinates of each seat are provided through a simple tabular structure. The generic nature of the input allows us to consider different vehicle types, e.g., school buses and airplanes.

#### Decision Variables

In mathematical programming, decision variables represent the desired outputs from the model. In our MIP model, the set of decision variables represents whether seats are occupied by any of the passengers. Mathematically, the decision variable capturing whether a given seat $j$ is occupied by a passenger is represented as follows:

\begin{align*} x_{j} = \begin{cases} 1 & \text {if a passenger is assigned to seat $j$}\\ 0 & \text {otherwise.} \end{cases}\end{align*}

Note that the number of seats, $m$, is defined by the provided layout. Furthermore, passengers are assumed to be homogeneous, i.e., there is no reason to treat passengers differently given the physical distancing requirement between any pair of passengers should be no less than the specified threshold. Thus, we do not introduce a subscript to distinguish between the different passengers.

#### Objective Function

In this optimization setting, the objective is to maximize the number of seats under specified physical distancing constraints. Mathematically, this can be represented as:

\begin{equation*} \max \sum _{j=1}^{m} x_{j}\end{equation*}

#### Constraints

Mathematical constraints are used to ensure that the resulting output is realistic and matches the requirements of the physical problem. Here, three sets of constraints are needed. The first constraint ensures that each $x_{j}$ is binary. To define the second and third constraints, we introduce new notation to capture whether two seats, say ( $a, \, b$), are allowed to be simultaneously occupied. Let $w_{a,b}$ be defined as follows:

\begin{align*} w_{a,b} = \begin{cases} 1 & \text {if the Euclidean distance between seats $a$and $b$} \\ & \text {is less than the physical distancing threshold}\\ 0 & \text {otherwise.} \end{cases}\end{align*}

Using the definition above, we limit $w_{a,b}$ to a binary outcome in the second constraint. The third constraint is used to prevent seats from being jointly occupied if they are too close as well as to allow them to be jointly occupied if they are further than the physical distancing requirements. Mathematically, these three constraints are formulated as follows:

(a)$x_{j}$ is binary $\forall j$.

(b)$w_{a,b}$ is binary, $\forall (a, ~b)$ where $a \neq b$.

(c)$x_{a} + x_{b} \le 2 - w_{a,b}, \forall (a, \,\,b)$ where $a \neq b$.

Recall that $w_{a,b}$ is equal to 1 if physical distance requirements are not met. Thus, the third constraint only allows for at most one seat in a $(a, b)$-pair to be filled when $w_{a,b} = 1$. Alternatively, the two seats may be occupied when $w_{a,b} = 0$.

### B. Algorithm for Seat Assignment With Boarding Order and Household Grouping

We next define a solution to assign seats to passengers by taking into account boarding order and the relationships among passengers. In short, the algorithm assigns seats from back to front, left to right, and it allows passengers from the same group (e.g., a family) to violate the physical distancing requirement. Algorithm 1 shows the pseudocode concerning our solution. As input, our algorithm requires an ordered set $S$ of 2-tuples representing the (x,y) coordinates of the $m$ available seats. The set’s ordering is defined by sorting the elements based on the $y$-axis values, followed by the $x$-axis values, both in descending order. This implies that the seats in the back are the first in $S$. Another input required by the algorithm is an ordered set $G$ of 2-tuples representing groups, where the first element in a 2-tuple is the group number defining the boarding order, and the second element is the total number of passengers in the group. Finally, the algorithm also requires a physical distance threshold $t \in \Re ^{+}$. The algorithm returns an ordered set $P$ that defines the group number (if any) associated with each seat. Formally, each element of $P$ can take on values from the set $\{\emptyset, 1, 2, {\dots }, z\}$, where $z$ is the total number of groups.

Algorithm 1 **Seat Assignment With Physical Distancing, Household Grouping, and Boarding Order**
**Input:**
$S = \{(x_{1}, y_{1}), (x_{2},y_{2}), {\dots }(x_{m}, y_{m})\}$
$G = \{(1, n_{1}), (2,n_{2}), {\dots }, (z, n_{z})\}$
$t \in \Re ^{+}$
**Output:**
$P = \{p_{1},p_{2}, {\dots }, p_{m}\}$
1:
$free\_{}seats \gets \{1, {\dots }, m\}$
2: 
3: **for** $(i, n_{i}) \in G$ **do**
4:
$seat\_{}index \gets free\_{}seats[{1}]$
5:
$group\_{}seats \gets \{\}$
6: 
7: **for** $j \in \{1, {\dots }, n_{i}$} **do**
8:
$P[seat\_{}index] \gets i$
9:
$free\_{}seats \gets free\_{}seats \setminus \{seat\_{}index\}$
10:
$group\_{}seats \gets group\_{}seats \cup \{seat\_{}index\}$
11:
$seat\_{}index \gets seat\_{}index + 1$
12: **end for**
13: 
14: **for** $a \in group\_{}seats \And b \in free\_{}seats$ **do**
15: **if** $D\left ({(x_{a},y_{a}), (x_{b}, y_{b})}\right) < t$ **then**
16:
$free\_{}seats \gets free\_{}seats \setminus \{b\}$
17: **end if**
18: **end for**
19: 
20: **end for**

The algorithm starts by defining that all seats are currently available (line 1). Next, the algorithm iterates over all groups (lines 3 to 20). For each group identified by the number $i$, the algorithm first retrieves the index of the first seat (furthest to the back) that is currently available (line 4). Moreover, it defines a variable that shall store information about all the seats to be occupied by members of group $i$ (line 5). Thereafter, the algorithm assigns each member $j$ of group $i$ to the first available seat (line 8); it removes the assigned seat from the set of available sets (line 9); it also adds the assigned seat to the set of seats occupied by group $i$ (line 10); and it finally updates the index of the first seat (furthest to the back) that is currently available (line 11).

The final step of the algorithm is to make some seats unavailable based on the physical distancing requirements. To do so, the algorithm goes through each pair of seats $(a,b)$, where $a$ represents a seat occupied by a member of group $i$ and $b$ correspondents to a seat that can, up to now, still be assigned. During this process, any available seat is marked as unavailable (line 16) if its Euclidean distance to an assigned seat is less than the physical distance threshold (line 15).

## III. Data Input/Output

To help practitioners to use our models in real-life, we developed a web application that naturally captures input data for the optimization models and returns the solution outputs in an easy-to-understand format. In what follows, we divide our discussion into two parts. First, we explain how a user can provide input data through our application. Next, we illustrate possible outputs from the optimization models.

### A. Inputting Data Into the Optimization Models

As we discussed in Section II, the required input data differ based on the choice of the optimization model. In particular, both models require a user to provide a social distance threshold and a layout of the vehicle. Moreover, Algorithm 1 requires a definition of groups. We next explain the format of such files and how a user can upload them to our application.

**FIGURE 2.**
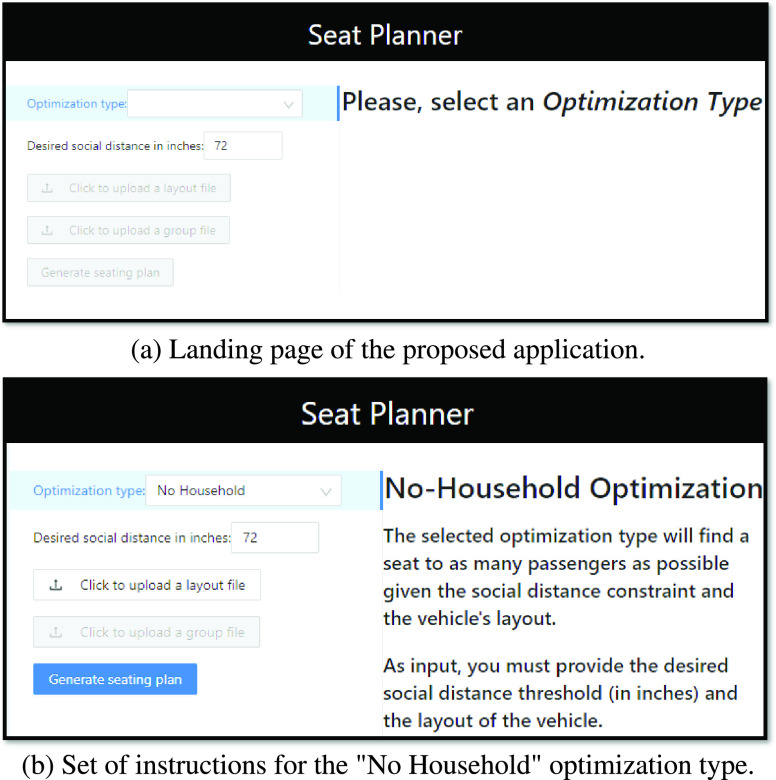
User interface of the proposed application.

Initially, a landing page asks the user to select an optimization type from a drop-down menu (see Fig. 2a). The drop-down menu provides two options, here named “No Household” and “Household”, which correspond to the models described in, respectively, Subsections II-A and II-B. Once the user selects his/her choice of optimization model, the application provides a list of instructions to help the user to upload the required data (see Fig. 2b). For both models, the user can define a desired social distance value by using a numeric input field. We set the default physical distance threshold to 72 inches (i.e., 6 feet or approximately 1.83 meters) based on the recommendations made by the Centers for Disease Control and Prevention (CDC) in the United States in [9]. Nevertheless, the user interface makes it easy to change such a value to accommodate for other policies/guidelines that can vary based on states and/or countries, e.g., the Massachusetts Department of Elementary and Secondary Education [21] has suggested using a distance of 3 feet in school buses.

**FIGURE 3.**
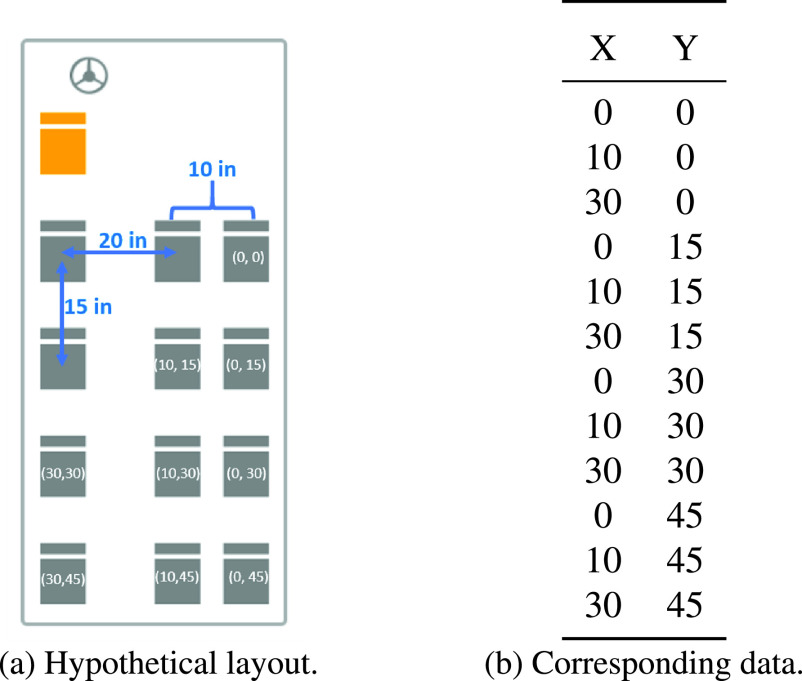
Input data concerning a vehicle’s layout.

The layout of the vehicle must be specified in a tabular format. Specifically, the user must define a table containing the (x,y) coordinates of every single seat. Such a table must be inside a file that follows either a Comma-Separated Values (CSV) or a Microsoft Excel Open XML Spreadsheet (XLSX) format. Fig. 3, which is part of the application’s instructions, provides an example of how the layout file should be formatted for the depicted (hypothetical) seating chart of a mini bus (see Fig. 3a). Note that the structure of the layout file (see Fig. 3b) is flexible to allow for applications involving different vehicles. For example, we present the results for applications involving school buses and airplanes in Section IV.

If, alternatively, the user selects “Household” as the preferred optimization type, then, besides the social distance threshold and vehicle’s layout, the user must also provide a file defining groups. Such a file must contain a table and be in either the CSV or the XLSX format. The underlying table has two columns. The first column (“GroupId”) defines a group’s number, where lower numbers are seated first. The second column (“NumberPassenger”) defines the number of members in a group. Table 1 illustrates the above format. We note that the order of the groups may impact the results from Algorithm 1, including the number of seated passengers. We assume that this input data can be obtained prior to using our application. For example, in school bus seating problems, the order reflects the students’ pickup order as determined by the route. Alternatively, in airplane seating, the order can be based on ticket price, passengers’ loyalty status, etc.

**TABLE 1:** Example of a Group-Related Input

GroupId	NumberPassengers
1	3
2	4
3	1
4	2
5	7
6	1

### B. Output Diagrams

**FIGURE 4.**
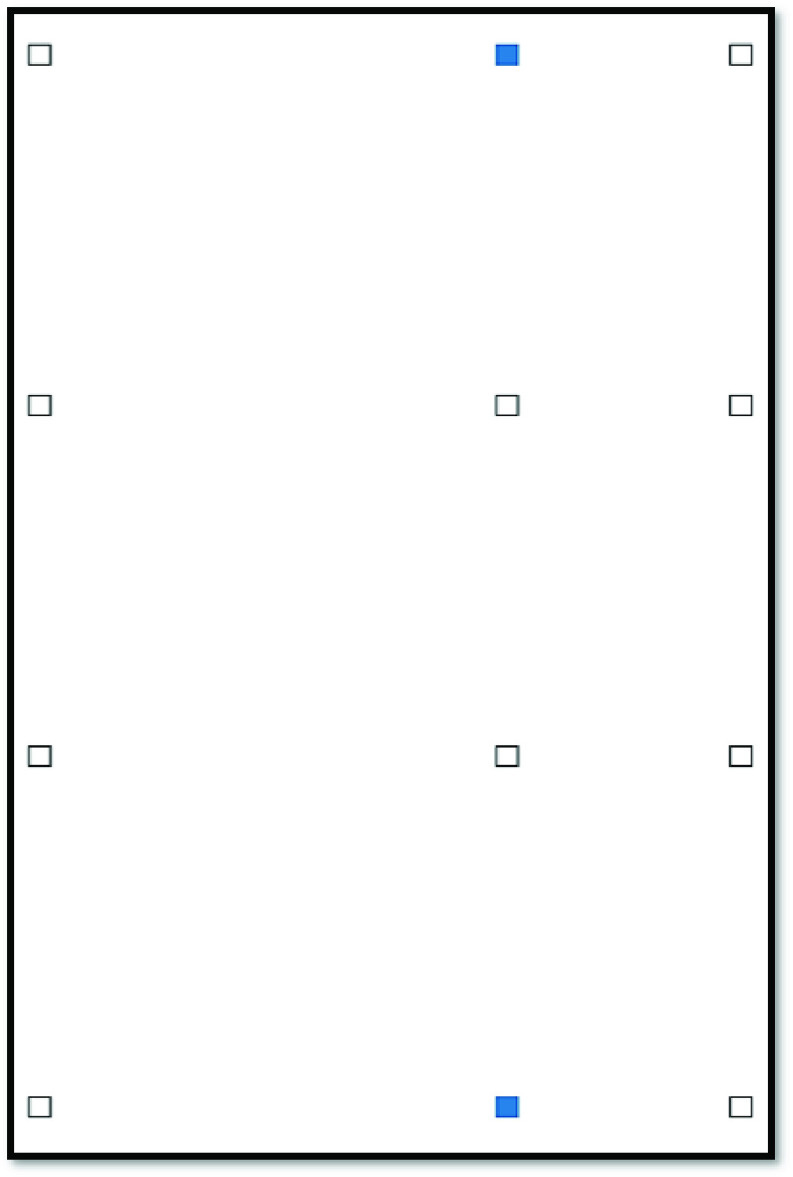
Sample output for the “No Household” model.

As Fig. 1 suggests, the proposed application produces an output after ingesting input data and performing the appropriate calculations. The output, currently implemented as a Portable Document Format (PDF) file, provides a visual depiction of the resulting seating arrangement. For the “No Household” optimization type, we adapt the approach presented in [15] to depict the model’s solution, where white squares represent unoccupied seats and blue squares represent occupied seats. For illustration purposes, Fig. 4 shows the output after applying the “No Household” (MIP) model on the mini bus example of Fig. 3 with a required physical distancing of 36 inches (3 feet or approximately 92 centimeters). It is noteworthy that only 2 of the 12 bus seats can be occupied in this example. The presented solution, thus, represents one of multiple optimal solutions, where two seats can be assigned while abiding by the required physical distancing constraints.

**FIGURE 5.**
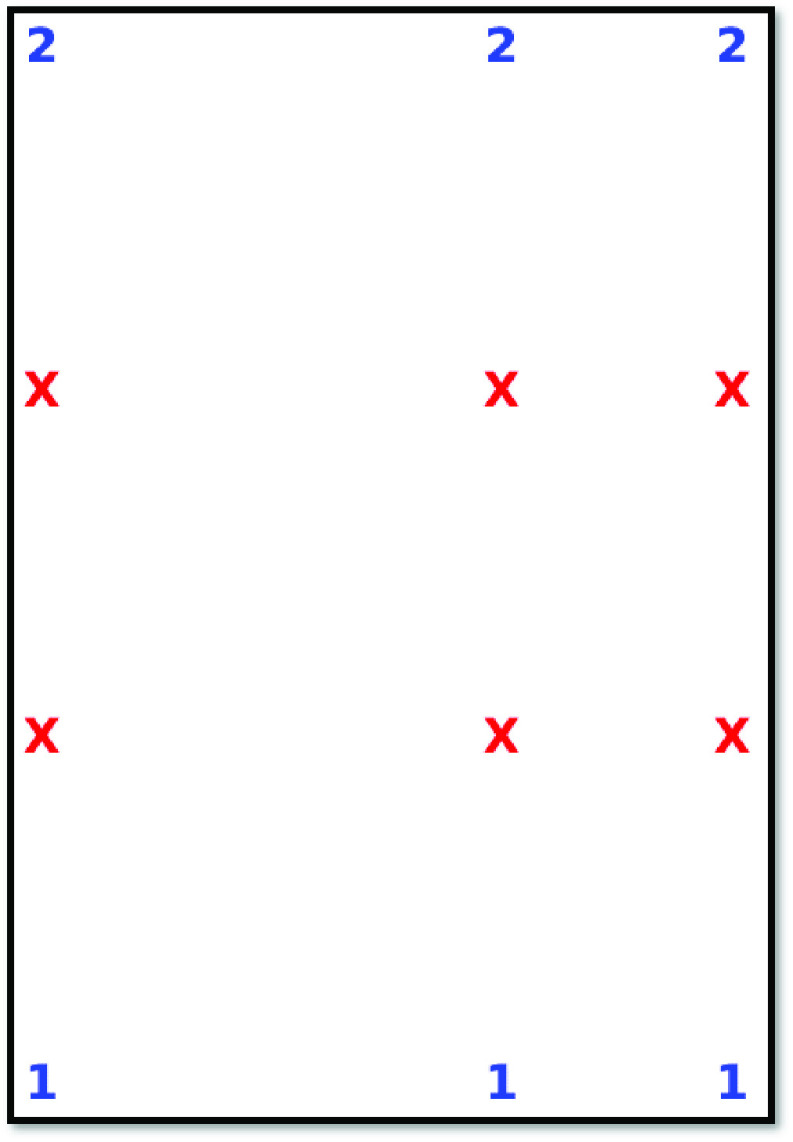
Sample output for the “Household” model.

For the “Household” model, the output diagrams are presented in a different format since they must specify where members of different groups are seated. The ‘X’ symbol denotes a particular seat is empty, whereas a number greater than zero means that a specific seat is assigned to a passenger from a group represented by that number. Finally, the value 0 (zero) means that a seat has been neither assigned to a passenger nor blocked due to social distancing requirements. Fig. 5 shows the output for the “Household” model applied to the mini bus example of Fig. 3, with grouping information from Table 1, and a minimum physical distancing between groups of 36 inches (≈ 91 cm). It is noteworthy that allowing for groups implies a higher number of seated passengers in this setting. We return to this point in the next section.

**FIGURE 6.**
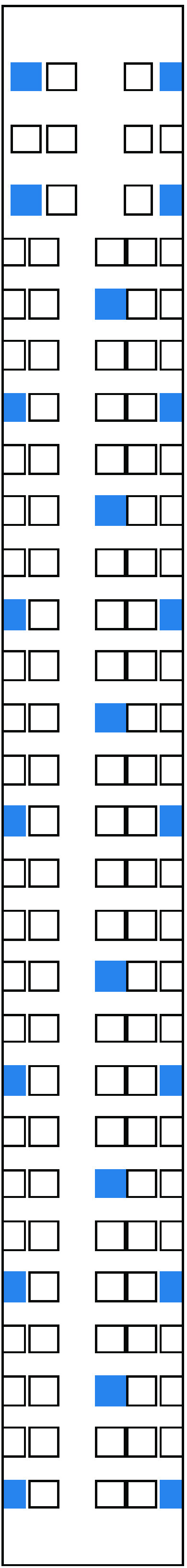
Sample output for an Airbus A220 seating plan.

We highlight the versatility of our application and its potential uses with an additional example. Specifically, we consider a typical layout of an Airbus A220 plane [22], where the MIP model is used to obtain a feasible seating plan when relationships are not considered and at least 6 feet of distancing is required. Fig. 6 shows the resulting output, where one can see, for example, the differences in layout and seating arrangements between the first class and the economy class.

To summarize, the seating plans generated by our proposed solution and illustrated above can be used to plan the capacity of transportation vehicles given physical distancing requirements. This will help the operators of these vehicles to efficiently and safely provide their services for as many people as possible given the nature of the underling domain, e.g., whether ordering and grouping are possible and/or desirable.

## IV. Computer Experiments

We next highlight the effectiveness of the proposed models and application when developing and presenting optimal seating arrangements for two types of transportation vehicles, namely school buses and airplanes.

### A. Public School Buses

Type “D” school buses are some of the most commonly used school buses in the United States. These buses have a maximum capacity of 90 students, but most operate with a 50-60 passenger capacity [23]. A school bus route designated for 54 passengers typically consists of passengers from approximately 36 separate households [24]. To incorporate potential variation in the household memberships of passengers across different routes, we generated 10 simulations of household membership for 5 different bus sizes. The number of passengers for a given group was generated using bootstrap sampling, with the group sizes set based on the following probabilities: (a) 40% for groups of 1 passenger, (b) 40% for groups of 2 passengers, (c) 15% for groups of 3 passengers, and (d) 5% for groups of 4 passengers. The code to create groups is available in the repository listed in the appendix.

In Table 2, we document the results from our experiment. The size of the bus, i.e., number of passenger seats, is captured in the first column. In the second column, we document the resulting 6 feet capacities as determined by the *Kentucky Department of Education* [13]. Note that their solution provides only the capacity, which needs to be translated into an actual seating arrangement by the school bus operators/schedulers. We note that our application can be adapted to generate an actual seating plan following the guidelines in [13]. In the third column, we provide the results from our MIP model. Finally, the fourth column provides the average number of seated students when considering grouping, as computed from the 10 simulated examples by Algorithm 1. All the fractions in the fourth column are rounded down.

**TABLE 2:** Results of the Numerical Experiment for School Buses and a 6-ft Minimum Physical Distancing Requirement

Bus Capacity in Persons (P)	KY Guidelines per [13] in P (% occupancy)	MIP Solution in P (% occupancy)	Heuristic Solution in P (% occupancy)
34P	4P (11.8%)	4P (11.8%)	5P (14.7%)
52P	6P (11.5%)	6P (11.5%)	8P (15.4%)
66P	7P (10.6%)	8P (12.1%)	10P (15.2%)
72P	8P (11.1%)	8P (11.1%)	11P (15.3%)
84P	9P (10.7%)	10P (11.9%)	13P (15.5%)

From Table 2, there are two general observations to be made. First, the results from the MIP solution are exactly the same as those provided in [13], with the exception of the 84-passenger bus solution, where our model suggested seating 10 students, as opposed to 9. Second, Algorithm 1 provides substantial efficiency gains in seating when compared to the MIP solution. In particular, the difference in the number of seated passengers varied between 25% (34P bus) and 37.5% (72P bus). Thus, this simplified experiment highlights the importance of considering household solutions in the context of the school bus transportation problem. Finally, we note that our application allows one to quickly generate solutions. Specifically, the average running time, including computations and printing out the diagrams, was 3.14 and 3.63 seconds for the MIP model and Algorithm 1, respectively.

### B. Commercial Airplanes

Compared to school buses, a greater variety of both vehicle layouts and passenger relationships exists for commercial airplanes. Next, we show how our models and application can be used to assign seats for prototypical layouts of Delta Air Lines Airbus A320 and Boeing 737 airplanes. To consider the effect of including household relationships, we generated 10 random ordered groups by using the following proportions, which are slightly adjusted from those used in the school bus example: (a) 30% of groups included 1 passenger, (b) 25% of groups included 2 passengers, (c) 20% of groups included 3 passengers, (d) 15% of groups included 4 passengers, (e) 5% of groups included 5 passengers, and (f) 5% of groups included 6 passengers.

Table 3 demonstrates the performance of our models in providing the optimal seating arrangements for multiple aircraft types and passenger groupings, with physical distancing of 3.3 feet as well as 6 feet. We contrast our solutions against the solutions by Salari *et al.* [11], who in turn captured how to maximize the safe loading of passengers while ensuring that no passengers are seated closer than a 3.3 feet distance from other passengers. We note that Salari *et al.* [11] only considered one configuration of the A320 aircraft, which is slightly different than the typical A320 aircraft layouts in the U.S. [25], [26], e.g., the former layout has no first class and premium economy seats. Thus, we have chosen to incorporate two layouts for the A320 aircraft. The first corresponds to Delta Air Lines’ layout for the A320 aircraft [25], [26], and the second corresponds to that presented in the work by Salari *et al.* [11]. Additionally, we incorporated one layout for the Boeing 737 aircraft.

**TABLE 3:** Results of the Numerical Experiment for Commercial Airlines With Varying Physical Distancing Requirements

Physical Distancing Threshold	Aircraft Type/Layout	Salari et al. [11] Solution (% occupancy)	MIP Solution (% occupancy)	Heuristic Solution (% occupancy)
3.3 ft.	A320	–	62P	76P
	(Delta [26])	–	(38.8%)	(47.5%)
	A320	30P	40P	46P
	(per [11])	(25.0%)	(33.3%)	(38.3%)
	B737	–	40P	47P
		–	(33.3%)	(39.2%)
6 ft.	A320	–	26P	47P
	(Delta [26])	–	(16.3%)	(29.4%)
	A320	–	20P	29P
	(per [11])	–	(16.7%)	(24.2%)
	B737	–	14P	29P
		–	(11.7%)	(24.2%)

From Table 3, there are two results that should be highlighted. First, our MIP solution seats 10 extra passengers when compared to the solution by Salari *et al.* [11, as depicted in their Figure 7 (right)]. We hypothesize that this variation could be attributed to the identification and adoption of a local maximum as the optimal value by those authors. Second, when household groupings are considered, the use of Algorithm 1 presented substantial efficiency gains, which varied between 15% (3.3 feet $\times $ A320 per [11]) and 107% (6 feet $\times $ Boeing 737). Moreover, the efficiency gains brought by Algorithm 1 in relation to the MIP model were uniformly higher for the 6 feet solutions. This highlights that the impact of household grouping becomes more substantial with more stringent physical distancing requirements. This result is intuitive since seating household members is not influenced by an increase in the overall physical distancing threshold.

## V. Conclusion

To assist with the unprecedented challenges and restrictions faced by single-destination public transit operators, this paper highlights how prescriptive analytics can be used to assign passengers to seats while accounting for different social distancing requirements. Overall, this study makes three major contributions. First, we have relaxed the implicit assumption of a singular physical distance requirement made by existing optimization routines [11], [14]–[16], which do not explicitly differentiate between passengers from the same and different households. From our experiments, we have documented the capacity gains that can be achieved when accounting for household relationships with different mixes of singular and within-same-household passengers. Second, our proposed algorithm allows for combining both the group-based seat assignment problem with an ordered, back-to-front boarding/loading policy. This enables us to tackle two typically distinct problems in the literature, leading to efficiency gains while improving the overall safety through reducing the likelihood of virus transmission. Third, this study bridges the gap between operations management/data analytics research and practice by presenting an example of how research can be packaged into an application open to the public. In particular, our currently running web application allows mass transit operators with limited resources to run “what-if” analyses and/or develop seating charts that accounts for different/changing social distance requirements. This is especially important for school bus coordinators in the U.S., who often resort to manual methods for assigning pupils to bus schedules/routes [20].

As with any study, there are limitations that need to be highlighted. For example, we have not considered application scenarios where practitioners can control the order by which passengers can be seated/picked. While our MIP model presents an upper limit to the efficiency that can be achieved by existing formulations that do not consider household groupings or boarding order, our model is nonetheless suboptimal in scenarios where the order by which passengers are picked/seated can be dynamically changed. This presents an opportunity for future research. We anticipate that one potential solution is to formulate the above scenario as a graph, where the nodes represent potential passengers and the arcs represent the social distancing requirement between the different nodes/passengers. Other approaches should be evaluated on the basis of reducing the computational complexity while ensuring the optimality of the attained results.

## Appendix Supplementary Materials

The source code of our application is available at *https://github.com/arthurgcarvalho/COVID-19_Seat_Planner* under the Creative Commons Zero (CC0) 1.0 Universal license. The optimization component was developed in Python. The graphical interface and server were written in, respectively, JavaScript and Node.js. A public, running application is available at *http://seatplanner.fsb.miamioh.edu/*.
